# Prioritizing research needs and opportunities at the intersection of implementation science and engagement science

**DOI:** 10.1186/s43058-024-00617-5

**Published:** 2024-07-18

**Authors:** Aubrey Villalobos, Elizabeth Reynolds, Sean N. Halpin, Sara R. Jacobs, Holly L. Peay

**Affiliations:** 1 Division of Cancer Control and Population Sciences, National Cancer Institute, 9609 Medical Center Dr., Bethesda, MD 20892 USA; 2 RTI International, 3040 E Cornwallis Rd, Research Triangle Park, NC 27709 USA

**Keywords:** Community engagement, Engagement processes, Engagement outcomes, Implementation collaboration, Health equity, Research opportunities, Research priorities, Research needs

## Abstract

**Background:**

There has been increased attention to the need for, and the positive impact of, engaged or participatory science in recent years. Implementation scientists have an opportunity to leverage and contribute to engagement science (ES) through the systematic integration of engagement into implementation science (IS). The purpose of this study was to gather information from researchers and others to develop a prioritized list of research needs and opportunities at the intersection of IS and ES.

**Methods:**

We conducted three Zoom-based focus groups with 20 researchers to generate a list of unmet needs, barriers, and to describe normative themes about use of ES and IS. Then a panel of nine experts in IS and/or engagement ranked the needs and barriers using a survey and met via a Zoom meeting to discuss and generate research opportunities and questions, with reference to the focus group outputs.

**Results:**

Respondents and experts concurred on the importance of engagement in IS. Focus group participants reported 28 needs and barriers under the themes of 1) need for best practice guidance related to engagement processes and outcomes and 2) structural barriers to integrating ES in IS. The expert panel prioritized six structural barriers and four barriers related to generating best practice guidance, with corresponding recommendations on research opportunities. Example research opportunities related to engagement processes included: define “successful” engagement in IS contexts; adapt engagement tools and best practices from other disciplines into IS. Example research opportunities related to outcomes included: assess the impact of engagement on IS outcomes; examine engagement practices that lead to optimal engaged research. Example research opportunities related to structural barriers included: leverage research evidence to create structural changes needed to expand support for engaged IS; examine factors that influence institutional buy-in of engagement in IS.

**Conclusions:**

Research needs exist that relate to engagement processes, outcomes, and structural barriers, even for scientists who value engaged research. Expert panelists recommended sequential and reinforcing research opportunities that implementation and engagement scientists can tackle together to advance both fields and health equity. Future work should assess insights from broader invested parties, particularly patients and community members.

Contributions to the literature

- This study responds to calls in the literature to increase community and partner engagement/participation by outlining research-related needs (e.g., recommendations, training, and structural changes) and scientific opportunities for integrating the study of engagement into dissemination and implementation research.
- These findings contribute to a gap by providing specific recommendations for how implementation scientists and researchers with engagement expertise can collaborate to advance critical questions relevant to both fields.
- These findings also speak to funders and research administrators, highlighting the unresolved barriers and structural changes needed to enable more engaged or participatory research, especially in partnership with underrepresented communities experiencing disparities.

## Background

Implementation science (IS) is the study of methods to promote the adoption and integration of evidence-based practices, interventions, and policies into routine healthcare and public health settings to improve the impact on population health [1]. As a field, IS benefits from engagement with healthcare and public health decision makers as well as real-world implementers and end users of evidence-based practices, interventions, and policies [2]. Recent evidence suggests that implementation scientists frequently conduct engaged or participatory research, though the level or depth of engagement varies [3].

IS professionals have called for increased focus on integrating engagement in IS in conjunction with an increasing focus on health equity and health disparities [4–6]. A 2023 scoping review by Gustafson et al. identified twelve IS theories, models, and frameworks (TMFs) with an equity focus [7]. Engagement of communities experiencing disparities was emphasized in most of them, particularly during implementation planning phases. Likewise, Wilkins et al. (2023) recently outlined how community-engaged research is essential to addressing health inequities [8]. Research focused on equity and disparities demands engagement with populations who are often marginalized and under resourced, to foster trust and support community empowerment and self-determination. Ramanadhan et al. (2023) provide a review of participatory IS to advance health equity that includes a rationale for this type of research, a framework for integrating participatory approaches into IS TMFs, as well as important considerations for how to do this work well [9].

Outside of IS, there is a long, rich history of engaged research that includes various models and approaches for engaging different groups in the pursuit of optimal opportunities for individuals or populations to attain the best health possible (i.e., health equity). Perhaps the most well-known because of its long history is Community Based Participatory Research, or CBPR. CBPR, as defined by the Kellogg Foundation, “equitably involves all partners … with a research topic of importance to the community with the aim of combining knowledge and action for social change to improve community health and eliminate health disparities” [10]. The Patient-Centered Outcomes Research Institute (PCORI) is a major funder of engaged healthcare research, including CBPR, and their model requires “meaningful involvement of patients, caregivers, clinicians, and other healthcare stakeholders throughout the entire research process—from planning the study, to conducting the study, and disseminating study results” [11]. However, several consistent barriers and challenges to conducting this type of highly engaged research have been documented, including the increased level of effort, time, and expertise in engagement methods required [9]. Further, not all IS studies require the same level of participation or engagement from the community or other partners, depending on the focus of the research. Therefore, other approaches and strategies may be most appropriate at intermediate (cooperation, collaboration) and minimal (outreach and education) levels of engagement [12].

There is a large body of evidence around the benefits of community engagement. Ramanadhan et al. 2023 highlight both instrumental (e.g., more relevant research, better understanding of local context) and normative (e.g., enhanced justice, inclusion, and equity) motivations for engaged research [9]. A 2020 meta-review of community-engaged scholarship by Ortiz et al. examined 100 reviews of research, 55 of which reported on partnership or health outcomes. They concluded that evidence largely supports community-engaged research contributing to improved health and health equity, as outlined in the CBPR conceptual model [13]. In addition, a qualitative analysis of the impact of engagement in 58 PCORI-funded studies found that researchers and their partners alike viewed engagement as worthwhile and noted it influenced the design and execution of the studies and was integral to the success of the research [14]. Combined with IS, engaged research approaches have great potential to move closer to health equity [15].

Given the prevalence of engagement activities in IS, it could be an opportune marriage to incorporate unanswered engagement-related scientific questions and measures into IS studies. Distinct from engaged research that uses various techniques for enabling input and participation of non-researchers, engagement science (ES) examines the methods and outcomes of engagement to build an evidence base for the value and quality of engagement [16–18]. As such, the 15th Annual Conference on the Science of Dissemination and Implementation included a forum titled, “Research Opportunities at the Nexus of Engagement Science and Implementation Science” [19]. Twenty-four attendees worked in small groups to identify important and opportunistic engagement-related scientific questions that could be answered through IS. Building upon this initial forum and brainstorming activity, the present study reports on subsequent data collection and activities to develop a prioritized list of scientific needs and research opportunities for implementation scientists and funding agencies to consider.

## Methods

We report on a series of focus groups about use of ES and IS, followed by discussion with expert panelists [20]. The purpose of the exploratory focus groups was to generate a diverse list of unmet needs, barriers, and to describe normative themes from the perspectives and experience of a multi-discipline group of participants. The purpose of the expert panel was to deliberate with the study team to structure and organize research opportunities and priorities for integrating ES and IS, using the needs and barriers generated from the focus groups as a starting point. This multi-step process was adapted based on several previous efforts to generate research priorities in other fields [21–23].

### Focus groups

#### Participants

We applied purposive sampling techniques to recruit focus group participants from a list of 55 ES and IS researchers using published literature and professional contacts [24]. Targeted participants had a mix of expertise in ES and/or IS, and a range in number of years of experience. We emailed a prescreen survey to the list of prospective participants and ES and IS professional listservs to identify interested individuals and let them indicate their expertise.

#### Procedures

In advance of the focus groups, participants were provided a series of suggested background readings and definitions related to ES and IS. Three Zoom-based focus groups were conducted in August 2023 and lasted about 90 min, with five to nine participants each. Focus groups were facilitated by the senior investigator (HP) with another investigator (ER) using a structured template to capture notes [25]. The discussion prompts followed the topic areas described in Table 1. Participants were asked to provide input on barriers and needs related to integrating ES into IS. Participants were informed that an expert panel would subsequently convene to aggregate research priorities informed by the focus group data.

**Table 1:** Focus group topic areas

ENGAGEMENT VALUE. Discuss engagement as a conceptual or normative approach to implementation science (i.e., why to use engagement in implementation science)
ENGAGEMENT PROCESSES. Discuss unmet needs and research opportunities about processes (i.e., how to incorporate engagement in implementation science)
ENGAGEMENT OUTCOMES. Discuss unmet needs and research opportunities related to how well we conduct engagement (i.e., defining engagement metrics/outcomes and assessing the impact of engagement)

#### Analysis

Initial focus group analysis was conducted by SNH using a rapid analysis approach to summarize the comments into specific themes and subthemes [26]. Next, using consensus-based revisions involving HP, SJ, SNH, and ER, we converted these themes and sub-themes into a list of 28 specific needs and barriers.

### Expert panel

#### Panelists and process

HP and AV selected and invited 14 individuals with notable scientific leadership and publications in ES and/or IS to serve on the expert panel, with input from the study team. The expert panel provided their feedback in two modes: completing a ranking activity and participating in one of two 90-min Zoom-based meetings. Participants ranked 100 randomly generated pairs of needs and barriers from the list of 28 identified by the focus groups, using an online-asynchronous free stack ranking tool OpinionX (https://www.opinionx.co/) (Fig. 1). We used the resulting item rankings to initiate the expert panel discussions. HP moderated the discussions, and ER and SNH took notes. The discussion began by HP requesting the experts generate research opportunities associated with the top ten ranked needs and barriers, though the discussion was not strictly limited to that list.

**﻿Fig. 1:**
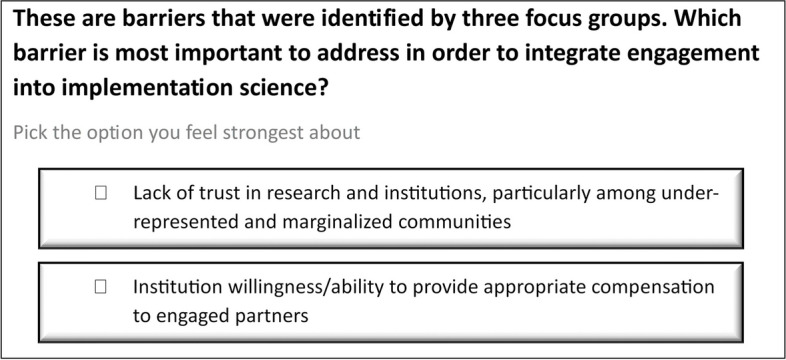
Ranking activity example

#### Analysis

SNH used a rapid analysis approach to sort and refine the expert-generated research opportunities and map them to the focus-group generated barriers and needs [26]. Some barriers generated multiple research opportunities and a subset of research questions spanned multiple barriers. The resulting matrix was reviewed by SJ, who provided additional IS context, and then by SNH and ER for further refinement and interpretation. We used quotes (which came from either verbal or chat communication during the expert panels) to further contextualize each research opportunity and identify associated research questions. A summary document was emailed to panelists for member checking, and minor further refinements were made to the list of recommendations and opportunities based on feedback received.

## Results

### Focus groups

#### Focus group participants

The twenty focus group participants resided in the United States (*n* = 18), Nigeria (*n* = 1), or Norway (*n* = 1) and indicated they had expertise in either IS (*n* = 10) or both ES and IS (*n* = 10). Most participants (*n* = 11) identified their role as “professor.” Other roles included researchers (*n* = 4), project/program directors (*n* = 2), and post-doctoral student (*n* = 1).

#### Engagement value and approaches

Participants discussed the value of engaging with community members, patients, and other impacted parties to develop sustainable solutions to meaningful problems. Focus group participants spoke about engagement as a being *“a moral imperative if using taxpayer dollars”* with improved likelihood of *“achiev[ing] the best performance when we engage communities.”* Moreover, researchers felt that engagement was necessary for fostering trust and reciprocity. One researcher stated:

*“Engagement is about trying to figure out how we stop just pushing our results out and build the relationships so that we’re answering the questions that people want… at its best, engagement is about making sure that the product we’re creating is the product that the community needs.”*

Participants reflected on a range of engagement approaches (e.g., consultants versus project co-leads), the selection of which leads to differences in the impact of the engagement, the methods used, and the required resources. In addition, respondents described the different barriers and opportunities associated with the short-term compared to long-term engagement of partners (which builds strong relationships, capacity, and trust but requires investigator commitment to nurturing relationships between studies). Overall, our respondents viewed engagement as resource-intensive and requiring extra commitment and investment from the research team, yet they emphasized that the benefits of integrating ES into IS exceed the resources put in.

#### Needs and barriers related to integration of ES and IS

Focus group respondents generated a broad set of needs and barriers at the intersection of ES and IS categorized under the themes and sub-themes shown in Table 2 and discussed below.

**Table 2:** Needs and barriers related to integration of ES and IS by theme

Theme	Sub-Theme	Need or Barrier Related to Integration of ES and IS
Need for Best Practice Guidance Related to Engagement Processes and Outcomes	N/A	Investigators need clearly defined engagement goals and processes
		It is challenging for investigators to account for variability in engagement partner preferences
		Investigators need training/guidance on integrating engagement in existing implementation science theories, models, or frameworks
		Investigators need training/guidance on adapting engagement approaches to different settings
		There is a need to identify consensus approaches and measures to assess the process of engagement
		There is a need to identify consensus approaches and meaningful measures to assess the outcomes of engagement
		It is a challenge for investigators to determine appropriate and equitable compensation for engagement partners
		It is a challenge for investigators to decide who to ‘invite to the table’ for engagement
		It is a challenge to meter the right amount of input: Determining how much external/partner input is necessary and actionable
	Investigator-level Barrier	Investigators must have a commitment to relationship building for engagement to be successful
		Investigators must be able to train co-investigators/professional collaborators in engagement
		There are vulnerabilities in engagement that are caused by the inherent reliance on individual relationships between investigator and partner
	Engagement Partner-level Barrier	There is a lack of trust in research and institutions, particularly among under-represented and marginalized communities
		A barrier for meaningful participation of engagement partners is the need for training/education on research/technical aspects, without jargon
		The grant proposal and review process is unfamiliar to engagement partners
Structural Barriers to Integrating ES in IS	N/A	Real change requires institutional buy-in
		It is challenging for investigators to sustain community relationships after funding ends
		Investigators must set reasonable expectations of engagement partners in the context of the funded study (e.g., scope, timeline)
	Barriers Generated by the Research Institution	Conducting engagement is not incentivized within research or healthcare institutions
		There are institutional barriers to contracting with community organizations
		Institution willingness or ability to provide appropriate compensation to engagement partners is a barrier
		Institutional (including IRB) often have discomfort/lack of familiarity with engagement activities, particularly with marginalized communities
		Engagement expertise may not contribute to investigators’ professional advancement
		Investigators are required to go ‘above and beyond’ for engagement activities (such as extra time, unpaid effort)
		Institutions do not often teach engagement skills to their staff
	Barriers Generated by the Funding Institution	The barrier of project budget limitations—Engagement is resource-intensive
		The long grant review timelines and low chance of funding may negatively impact engagement relationships
		The barrier of limited project period of performance—Engagement takes time

#### Need for best practice guidance related to engagement processes and outcomes

A total of 15 barriers were categorized under this theme. These items reflected the need for consensus or evidence-based guidance on conducting engagement and measuring its impact. Most of the barriers reflect the need for practical “how to” guidance related to initiating, maintaining, and sustaining engagement. Examples include clearly defined engagement goals and processes, which engagement partners should be included, best practices for achieving shared goals across different types of engagement partners, engagement strategies to use at different stages of implementation and in different settings, and appropriate compensation for engagement partners. For example, when prompted to describe needs around processes, one focus group participant responded: *“How to listen first to hear community needs—how to create space for communities to share their perceived needs and priorities.”*

Respondents also reflected on the importance of consensus regarding meaningful ES outcome measures and integrated ES and IS TMFs. When describing how to measure impact of engagement, one focus group participant said, *“We often rely on frequency metrics like number of activities held, but it can be misleading if quality of those engagements is lacking.”* Another participant asked, “*Who defines what success looks like?”* Focus group participants noted the utility of an integrated model to support ES process evaluation and assessment of the impact of integrating engagement on implementation outcomes. As stated by one focus group participant, it is *“not one size fits all. One research opportunity is to document and understand how engagement is done in different settings for different groups.”* Two sub-themes consisted of barriers at the investigator and partner level, which all related to the need for guidance or capacity building on ES processes and navigating structural barriers.

##### Investigator-level Barriers

Respondents reported three investigator-level barriers to ES in IS. Two items relate to relationship building—one being the willingness and ability of the research team to develop and maintain relationships with engagement partners, and the other being the inherent effort and vulnerability stemming from the interpersonal relationships required to make engagement successful. Respondents described the importance of relationships among investigators and engagement partners to the establishment and maintenance of engagement. Investigators noted a need for guidance on facilitating engagement intensity and sustainability. Participants voiced particular concern about the maintenance of community-based projects. One focus group participant stated:

“I think about this in the community…I worry sometimes that some of my projects are one person away from basically just going out the window because they rely so much on these individual relationships. So how do you move past that? And I don’t know the answer, but I see that as a problem.”

A related concept was the need for guidance on activities that are effective at sustaining engagement with partners. Some respondents reflected on the professional and personal cost of using engaged research approaches, which may require more of investigators (e.g., work outside of standard hours, extra travel) while not being highly valued or recognized by some institutions. However, as noted above, many respondents also reported highly valued personal and professional benefits of conducting ES in IS.

##### Engagement Partner-level Barriers

Focus group members identified three engagement partner-level barriers. One of these barriers is mistrust of researchers and institutions, particularly among under-represented and marginalized communities. Respondents reported an imperative to engage with under-represented and distrustful communities, but also a need for guidance on how to build collaboration and trustworthiness across diverse research and engagement teams. The other barriers reflected the arcane vocabulary used in medicine and science and the unfamiliar and technical aspects of research and grant proposals. Respondents discussed the need for plain language materials that are accessible to partners so that all parties can provide meaningful input. Focus group members also described the need to familiarize engagement partners with the grant proposal and review process, the research process, and the structures and limitations that are imposed on IS by funding agreements, protocols, resource limitations, and regulations. Related to the critical need to translate technical information, a focus group participant described the parallel development of the IS approach and a guide for engagement partners:

“You know, for a community-based group…we kind of miss a gap in that it’s too conceptual. So even in some of the work that we’ve been doing with [institution name] we kind of think not only about an implementation blueprint, but then how can we create an adaptation guide where it’s very layperson friendly?”

#### Structural barriers to integrating ES in IS

The focus groups identified 13 structural barriers. Overall, respondents requested that institutions and funders remove roadblocks to successfully integrating ES in IS as well as reduce barriers to entry for engagement partners and investigators who are new to engagement. Under this domain were two subthemes.

##### Barriers generated by the research institution

Respondents described challenges associated with conducting engagement activities within a non-supportive or non-experienced institution and/or an environment where engagement is not incentivized. These challenges were described as procedural (e.g., institutional reticence to appropriately compensate engagement partners) and related to professional incentives (e.g., promotion). Institutional support for training was also needed to better prepare investigators; as one participant described, the need for ES *“intentionally being part of graduate and post doc training.”*

##### Barriers generated by the funding institution

Respondents reported several procedural or instrumental barriers generated by funding institutions. For example, traditional grant timelines and budgets do not account for the specific needs of engaged research, which is more time- and resource-intensive. Respondents also described ways in which typical grant processes and life cycle negatively impacted some aspects of investigator and engagement partner relationships, particularly with partners who are less familiar with research. Examples include attempting to maintain relationships once funding ends (in anticipation of future engaged research) and that long grant review timelines and low chance of funding was related to challenges with establishing community partnerships whose availability may shift once a study is funded or who may be less inclined enter into new partnerships later if a study is not funded initially. One focus group participant stated:

“How can we describe some of our wonky funding systems in academia with our engaged partners? Like I can work months with a partner to write a grant. We want to do this work, we get everyone excited and then six months later, I’m like, ‘well, sorry, we didn’t get funding.’”

While focus group participants agreed that structural barriers de-incentivized future engaged IS, they also agreed that this category was only minimally amenable to meaningful research questions.

### Expert panel

#### Expert panelists

Of the 14 invited, 9 accepted the invitation to serve on the expert panel. Expert panel participants had expertise in ES and/or IS and are listed in Table 3.

**Table 3:** Expert panelists

1. Ana Baumann, Washington University in St. Louis
2. Mabel Crescioni, Patient-Centered Outcomes Research Institute (PCORI)
3. Elizabeth Cope, AcademyHealth
4. Monica Pérez Jolles, University of Colorado
5. Brian Mittman, Kaiser Permanente
6. Rogério M. Pinto, University of Michigan
7. Suz Schrandt, ExPPecT
8. Andy Tan, University of Pennsylvania
9. Vetta Sanders Thompson, Washington University in St. Louis

#### Ranking of focus-group generated needs and barriers

The expert panel ranked the 28 needs and barriers in order of importance and the top 10 were used to initiate a discussion of research opportunities (Table 4). Six of the top 10 were structural barriers and the remaining 4 were needs related to generating best practice guidance for engagement processes and outcomes.

**Table 4:** Top ten needs and barriers as ranked by expert panel

Rank By Expert Panel	Need or Barrier	Theme
1	Investigators need clearly defined engagement goals and processes	Need for Best Practice Guidance Related to Engagement Processes and Outcomes
2	Conducting engagement is not incentivized within research or healthcare institutions	Structural Barriers to Integrating ES in IS: Barriers generated by the research institution
3	The barrier of project budget limitations—Engagement is resource-intensive	Structural Barriers to Integrating ES in IS: Barriers generated by the funding institution
4	There are institutional barriers to contracting with community organizations	Structural Barriers to Integrating ES in IS: Barriers generated by the research institution
5	It is challenging for investigators to account for variability in engagement partner preferences	Need for Best Practice Guidance Related to Engagement Processes and Outcomes
6	The long grant review timelines and low chance of funding may negatively impact engagement relationships	Structural Barriers to Integrating ES in IS: Barriers generated by the funding institution
7	Investigators need training/guidance on integrating engagement in existing implementation science theories, models, or frameworks	Need for Best Practice Guidance Related to Engagement Processes and Outcomes
8	Investigators need training/guidance on adapting engagement approaches to different settings	Need for Best Practice Guidance Related to Engagement Processes and Outcomes
9	Institution willingness or ability to provide appropriate compensation to engagement partners is a barrier	Structural Barriers to Integrating ES in IS: Barriers generated by the research institution
10	Real change requires institutional buy-in	Structural Barriers to Integrating ES in IS

#### Expert panelists’ discourse about research opportunities and priorities

Table 5 presents the expert panel recommendations on research opportunities important for increasing ES in IS organized around three areas, which stemmed from the focus group themes: (1) engagement processes, (2) engagement outcomes, and (3) structural barriers.

**Table 5:** Expert panel recommendations and research opportunities

**Recommendations on Research Opportunities Related to Engagement Processes**
A. Conduct research to define engagement, including: • Define engagement goals and “successful” engagement in IS contexts • Define meaningful outcomes to measure impacts or outcomes of engagement: o based on context/objectives/type of engagement used o outcomes of importance to research teams and institutions o outcomes of importance to engagement partners and communities B. Determine which engagement theories, models and/or frameworks (TMFs) are most useful for different IS needs or objectives. Integrate engagement constructs into IS TMFs C. Generate evidence-based recommendations and training materials • Define levels of engagement and strategies associated with each level/depth of engagement—the ‘core function and forms’ of engagement in IS • Adapt engagement tools and best practices from other disciplines into IS • Generate specific guidance drawn from ES and tailored for IS on: o building and supporting a diverse research team o creating relationships/partnerships—who to engage and when o characterizing the governance structure and team roles and responsibilities o defining shared norms and priorities across the research team and engagement partners o fostering positive, strengths-based approaches o developing objectives, aims, proposal/protocols/procedures along with engagement partners o maintaining meaningful engagement throughout project implementation, data analysis, interpretation, and dissemination o supporting long-term relationships
**Recommendations on Research Opportunities Related to Outcomes**
D. Assess the impact of engagement on IS outcomes: • Evaluate situational fit: assess outcomes based on the program/project objectives and the type and level of engagement employed • Evaluate the ability of engagement processes to generate change in both implementation process and outcomes, particularly on uptake/adoption, acceptability, cost effectiveness, and sustainability E. Sample specific research questions about engagement to integrate into IS studies: • What are the engagement practices that lead to optimal engaged research (e.g., sustained relationships; shared values; impactful dissemination)? • Who is receiving invitations to engage in research and why are they receiving those invitations (compared to others)? • How can we enable multiple communication modes and channels to voice opinions or preferences? • What are effective ways to bring groups together to meet a shared goal? • How well are different partners engaged throughout the research process (i.e., true vs tokenistic engagement)? • What are effective, feasible, and cost-effective activities (such as when resources are limited) for sustaining engagement with communities and other partners? • What is the impact of the research/program context on effectiveness of engagement approaches? • What facilitators are associated with successful engagement? • How can we measure the effectiveness of team composition and leadership structure (multidisciplinary/multi-experience and inclusive of multiple invested parties) for engagement in research? How does the structure, expertise and composition of the research team affect engagement outcomes?
**Recommendations on Research Opportunities Related to Structural Barriers**
F. Leverage research evidence to create structural changes needed to expand support for engaged IS • Foster champions for this type of work • Build funds, evaluative components, and timeline expectations consistent with high-quality engagement into funding opportunities • Generate new approaches to amplify and reward engagement for investigators (e.g., adding a section to highlight engagement expertise in professional biosketches; opportunities for engagement to help promotion/tenure) G. Sample specific research questions about structural barriers to engagement in IS: • What factors influence institutional buy-in of engagement in IS? • To what extent does engaged research increase policy maker confidence in research findings and recommendations? • To what extent do grants that facilitate engagement (e.g., timelines, funding, expectation setting, etc.) improve outcomes? • What is the impact of engaged research on trust in health systems, particularly in minoritized communities? • What are the optimal strategies for training researchers, students, research partners, and institutional leaders to participate in, conduct, or support engaged research? When training researchers, how do we embed the “will” to do real engagement researchers? How to support trainees/trainings/lower barriers to entry for the “engagement-curious” researcher & community partner?
**Overarching Recommendations** • Robust evidence generation on ES outcomes is critical, though additional effort will be required to incentivize uptake and institutionalization of ES practices • Seek additional viewpoints, particularly focused on patient and community perspectives, into future efforts to refine needs and research priorities

Expert panelists agreed that there are needs and opportunities related to defining goals, processes, and outcomes of engagement and providing best practice guidance for conducting engaged IS. They also concurred that engagement must be suited to the context and objectives—there is not a one-size-fits-all approach. Panelists emphasized that data are needed to advance the field from practice-based guidance to evidence-based, and to demonstrate the impact of engagement ‘done well’ on implementation outcomes. Such evidence is needed to inform change and support adoption at the structural level. To be successful in achieving research opportunities, experts reflected on the importance of institutional support; employing committed multidisciplinary teams that include ES and IS experts; and meaningfully integrating invested and impacted parties (e.g., patients, community members, minoritized groups, clinicians and other ‘on the ground’ implementers) into the process, including in leadership roles.

Once engagement processes and outcomes are defined, expert panelists agreed that is critical to assess the impact of engagement on implementation outcomes. They also outlined several specific opportunities related to studying engagement processes and outcomes within IS studies. To facilitate this research, panelists endorsed using or adapting existing ES tools and best practices from other fields for IS studies, including a need for integrating ES into existing IS TMFs. These would need to be flexible enough to apply to a variety of settings and populations. Emphasis was placed on the need for integration rather than the creation of new TMFs, as one panelist stated, *“We don’t want a new model. Just help us figure out how to integrate within models we’re already familiar with and whether that’s an engagement or an implementation model.”* The desired updated TMFs were framed as “causal”, and could be used to determine how, why, when, and where to engage for the purpose of improving implementation. Panelists acknowledged the complexity of developing causal TMFs related to engagement, but agreed that it is a necessary step for providing intentional guidance in this area: *“this to not only thinking through the goals and processes, but you know, developing an explicit causal model, what are the precise causal mechanisms and effects of engagement on the desired outcomes once we’ve identified those desired outcomes.”*

Panelists agreed that structural changes are needed to make engagement more feasible, for engagement to be prioritized, and for engagement activities to be sustainable. They supported the idea that evidence generated from process and outcomes research could be used to reinforce the value of engagement and build the will for and guide necessary structural changes. Nevertheless, panelists agreed that structural level changes would be challenging but still needed to be addressed to maximize the impact of engagement-focused research.

Finally, expert panelists noted the importance of integrating additional viewpoints, particularly focused on patient and community perspectives, into future efforts to refine needs and research priorities. Panelists called out specific needs associated with engagement of minoritized populations that may require an intentional focus in IS research, engagement, and associated priority setting. 

## Discussion

Focus group participants and expert panelists echoed critical points found throughout the engagement literature about the positive value of, but challenges to, engaging partners and communities in research broadly and in IS in particular. A recent analysis of National Institutes of Health (NIH) funded IS grants found that 87% involved some degree of community or partner engagement [3]. However, most (60%) were categorized as outreach or consultation, the lowest levels on a continuum [12] of the extent to which a community can be involved in research [3]. This might stem from limited formal training and inexperience with engagement, which leads to a lack of sophistication in describing the approach, or a hesitancy to go deeper in the engagement with partners due to noted barriers. As evidenced in our study, there is clearly a desire to conduct engaged IS but needs and barriers remain in understanding what is being done currently and how to improve it. Six of the top ten needs and barriers ranked by the expert panel, not unique to IS, are structural barriers erected by funding agencies (e.g., grant timelines, budget limitations) and academic institutions (e.g., contracting and compensating partners, incentivizing engaged research). Another precursor need to enable integration of meaningful engagement in IS is evidence-based guidance on engagement processes and outcomes. Implementation scientists, in partnership with engagement scientists, can help generate this guidance by investigating the questions outlined by the panelists.

Of critical importance to advancing this work is the integration of engagement into IS TMFs and incorporation of engagement outcomes measures in IS studies. A 2021 review by Pinto et al. reported that only 20% of IS TMFs reviewed included the 5 community engagement constructs assessed and that few TMFs with community-related constructs has been introduced since 2009 [27]. This aligns with the panel recommendation that engagement and implementation scientists collaborate to determine which engagement TMFs are most useful for different IS needs or objectives and integrate engagement constructs into IS TMFs.

Despite the plethora of evidence of the importance of engaged or participatory research, some still diminish the largely qualitative evidence and question the impact of engagement [28]. Indeed, Ortiz and colleagues (2020) note in their review of reviews that synthesizing the community-engaged research literature was challenging due to the largely qualitative nature of research methods and reports. They encourage future research on the development of evaluation tools for community-engaged research and on tailored models or approaches for specific populations experiencing health inequities [13]. Similarly, expert panelists here recommended defining meaningful outcomes to measure the impacts or outcomes of engagement, assessing the impact of engagement on implementation outcomes, and they specified multiple specific research questions about engagement outcomes to integrate into IS studies. To facilitate a head start for investigators, the National Academy of Medicine’s Organizing Committee for Assessing Meaningful Community Engagement in Health and Health Care has outlined a conceptual model on the dynamic relationship between community engagement and outcomes and has begun to curate instruments and questions used in different contexts and communities to assess engagement [29, 30].

After years of funding research that required engagement with patients and others involved in healthcare, PCORI took a step toward addressing remaining critiques and skepticism about the value and outcomes of engagement with their Science of Engagement funding opportunity. First issued in 2022, this opportunity funds studies that build an evidence base on engagement in health research. The science of engagement is an emerging area of inquiry that moves beyond simply conducting engaged research to examine the engagement itself. PCORI’s funding opportunity focuses on two areas, with initial funding priority given to the first: 1) development and validation of reliable quantitative measures for engagement structure or context, processes, and outcomes and 2) developing and testing engagement techniques to generate evidence on the most effective engagement approaches, particularly for underrepresented populations [18, 31]. While this is promising in addressing some of the recommendations of the expert panel, other funders could also contribute to more rapidly moving the field forward by attending to recommendations on research opportunities related to the structural barriers outlined.

**Fig. 2:**
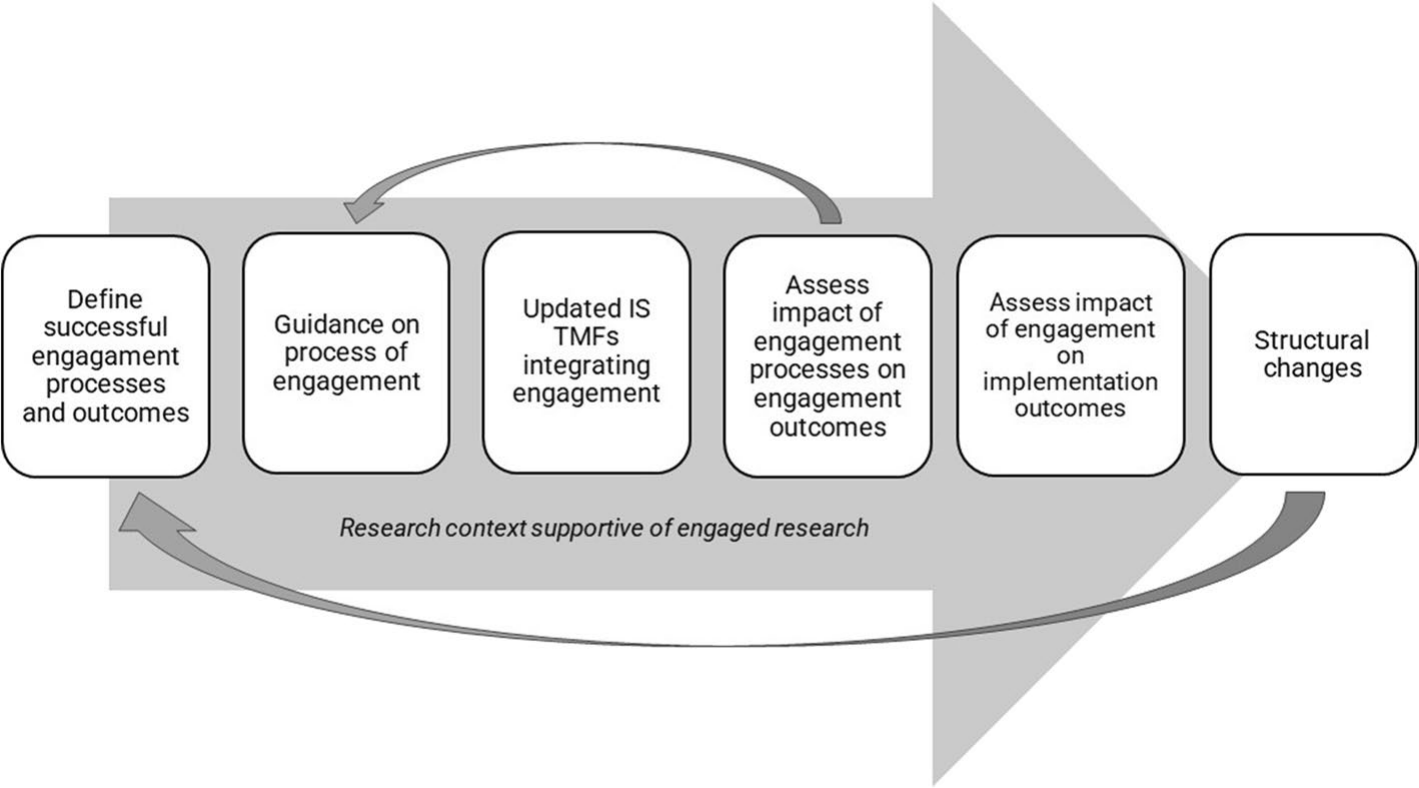
Sequential and reinforcing nature of ES in IS

Many of the research opportunities presented here are sequential and reinforcing (Fig. 2). Agreement about defining successful engagement processes and outcomes is a necessary initial step prior to conducting engaged IS that can generate evidence related to engagement effectiveness and the impact of engagement on implementation outcomes, which is needed to offer evidence-based guidance on engagement processes. This evidence in turn may be helpful to efforts to garner support for needed structural changes at research and funding institutions, which would enhance the research context in favor of supporting engaged research.

If achieved, structural changes would facilitate conducting more IS with ES integrated, thereby advancing both the sciences of implementation and engagement for improved population health outcomes and health equity. As an example, NIH’s Community Engagement Alliance (CEAL) has developed the CEAL Consultative Resource as a channel for sharing best practices to NIH-funded research teams wanting to apply principles of community-engaged approaches to address health disparities and ensure inclusion in research programs [32]. They offer 1:1 consultations as well as a growing library of evidence-based toolkits and videos on engagement best practices, including in many of the topics outlined in Table 5 item C; this in turn is enabling NIH-funded researchers to conduct high-quality engaged IS.

### Strengths and limitations

Strengths of this research prioritization study include the multiple sources of input beginning organically at the 15th Annual Conference on the Science of Dissemination and Implementation followed by focus groups with scientists with diverse professional and personal backgrounds, and a panel of senior scientists and experienced patient and community advocates. Limitations include that the needs and barriers identified differed by scope and scale (i.e., some are bigger problems than others), making them difficult to rank. Similarly, some of the recommendations on research opportunities were more specific than others. We had feasibility limitations in the number of focus groups that could be conducted; additional focus groups would likely have generated additional needs, barriers, and research opportunities. Finally, while our respondents and panelists came from a range of backgrounds, this study included only professional respondents with a preponderance of engagement scientists. Despite efforts to recruit participants and experts with perspectives from low- and middle-income countries (LMICs), only one focus group participant was from Nigeria and all expert panelists were U.S.-based. Our findings may not reflect the priorities of other parties, including patients, community members, clinicians, and those of researchers and communities in LMICs.

## Conclusions

The present study reports on the development of a collection of priority scientific needs and research opportunities related to engagement for implementation scientists and funding agencies to consider. Main conclusions are that research needs exist that relate to engagement processes, outcomes, and structural barriers, even for scientists who are bought in to the value of engaged research. Expert panelists recommended sequential and reinforcing research opportunities that implementation and engagement scientists can tackle together to advance both fields and health equity. Future work should assess insights from broader invested parties, particularly patients and community members.

## Data Availability

De-identified and aggregated findings of this study are available from RTI International upon reasonable request. Transcripts will not be made available given the nature of qualitative research and the difficulty in adequately de-identifying complete transcripts.

## Abbreviations

CBPR: Community-Based Participatory Research
CEAL: Community Engagement Alliance
ES: Engagement science
IRB: Institutional review board
IS: Implementation science
NIH: National Institutes of Health
PCORI: Patient-Centered Outcomes Research Institute
TMFs: Theories, models, and frameworks

## References

[1] National Cancer Institute. About Implementation Science 2022 updated August 04, 2022. Available from: https://cancercontrol.cancer.gov/is/about.

[2] Holt CL, Chambers DA. Opportunities and challenges in conducting community-engaged dissemination/implementation research. Transl Behav Med. 2017;7(3):389–92. 28884305 10.1007/s13142-017-0520-2 PMC5645293

[3] Villalobos A, Blachman-Demner D, Percy-Laurry A, Belis D, Bhattacharya M. Community and partner engagement in dissemination and implementation research at the National Institutes of Health: an analysis of recently funded studies and opportunities to advance the field. Implement Sci Commun. 2023;4(1):77. 37438834 10.1186/s43058-023-00462-y PMC10339604

[4] Baumann AA, Long PD. Equity in Implementation Science Is Long Overdue. Stanf Soc Innov Rev. 2021;19(3):A15–7.

[5] Brownson RC, Kumanyika SK, Kreuter MW, Haire-Joshu D. Implementation science should give higher priority to health equity. Implement Sci. 2021;16(1):28. 33740999 10.1186/s13012-021-01097-0 PMC7977499

[6] Shelton RC, Brownson RC. Enhancing Impact: A Call to Action for Equitable Implementation Science. Prev Sci. 2023;25(Suppl 1):174–89. 37878237 10.1007/s11121-023-01589-z PMC11133096

[7] Gustafson P, Abdul Aziz Y, Lambert M, Bartholomew K, Rankin N, Fusheini A, et al. A scoping review of equity-focused implementation theories, models and frameworks in healthcare and their application in addressing ethnicity-related health inequities. Implement Sci. 2023;18(1):51. 37845686 10.1186/s13012-023-01304-0 PMC10578009

[8] Wilkins CH, Miller ST, Richmond AN, Carrasquillo O. Community-Engaged Research - Essential to Addressing Health Inequities. N Engl J Med. 2023;389(21):1928–31. 37982404 10.1056/NEJMp2307774 PMC11088953

[9] Ramanadhan S, Aleman R, Bradley CD, Cruz JL, Safaeinili N, Simonds V, Aveling EL. Using Participatory Implementation Science to Advance Health Equity. Annu Rev Public Health. 2023;45(1):47–67. 10.1146/annurev-publhealth-060722-024251 PMC11251496 38109515

[10] Minkler M, Wallerstein N. Community Based Participatory Research for Health: Process to Outcomes. 2nd ed. San Francisco: Jossey Bass; 2008.

[11] PCORI. The Value of Engagement in Research 2018 updated April 24, 2023. Available from: https://www.pcori.org/engagement/value-engagement.

[12] Sanders Thompson VL, Ackermann N, Bauer KL, Bowen DJ, Goodman MS. Strategies of community engagement in research: definitions and classifications. Transl Behav Med. 2021;11(2):441–51. 32421173 10.1093/tbm/ibaa042 PMC8135186

[13] Ortiz K, Nash J, Shea L, Oetzel J, Garoutte J, Sanchez-Youngman S, Wallerstein N. Partnerships, Processes, and Outcomes: A Health Equity-Focused Scoping Meta-Review of Community-Engaged Scholarship. Annu Rev Public Health. 2020;41:177–99. 31922931 10.1146/annurev-publhealth-040119-094220 PMC8095013

[14] Maurer M, Mangrum R, Hilliard-Boone T, Amolegbe A, Carman KL, Forsythe L, et al. Understanding the Influence and Impact of Stakeholder Engagement in Patient-centered Outcomes Research: a Qualitative Study. J Gen Intern Med. 2022;37(Suppl 1):6–13. 35349017 10.1007/s11606-021-07104-w PMC8993962

[15] Wallerstein N, Duran B. Community-based participatory research contributions to intervention research: the intersection of science and practice to improve health equity. Am J Public Health. 2010;100(Suppl 1):S40–6. 20147663 10.2105/AJPH.2009.184036 PMC2837458

[16] Dungan R, Angove R, Cope E, Peay H. AcademyHealth, editor. 2019. Available from: https://academyhealth.org/blog/2019-01/engagement-science-introducing-inclusive-research-practices-potential-impacts. Cited 2023.

[17] Meissner P, Cottler LB, Eder MM, Michener JL. Engagement science: The core of dissemination, implementation, and translational research science. J Clin Transl Sci. 2020;4(3):216–8. 32695491 10.1017/cts.2020.8 PMC7348030

[18] PCORI. Advancing the Science of Engagement PCORI Funding Announcement -- Cycle 2 2022. 2022. Available from: https://www.pcori.org/funding-opportunities/announcement/advancing-science-engagement-pcori-funding-announcement-cycle-2-2022.

[19] AcademyHealth. Research Opportunities at the Nexus of Engagement Science and Implementation Science. 2022. Available from: https://academyhealth.confex.com/academyhealth/2022di/meetingapp.cgi/Session/32859.

[20] O’Brien BC, Harris IB, Beckman TJ, Reed DA, Cook DA. Standards for reporting qualitative research: a synthesis of recommendations. Acad Med. 2014;89(9):1245–51. 24979285 10.1097/ACM.0000000000000388

[21] Byrne M, McSharry J, Meade O, Lavoie KL, Bacon SL. An international, Delphi consensus study to identify priorities for methodological research in behavioral trials in health research. Trials. 2020;21(1):292. 32293510 10.1186/s13063-020-04235-z PMC7092577

[22] Kobeissi L, Nair M, Evers ES, Han MD, Aboubaker S, Say L, et al. Setting research priorities for sexual, reproductive, maternal, newborn, child and adolescent health in humanitarian settings. Confl Health. 2021;15(1):16. 33771212 10.1186/s13031-021-00353-w PMC7995567

[23] Norris E, Prescott A, Noone C, Green JA, Reynolds J, Grant SP, Toomey E. Establishing open science research priorities in health psychology: a research prioritisation Delphi exercise. Psychol Health. 2022:1–25. https://www.tandfonline.com/doi/full/10.1080/08870446.2022.2139830. 10.1080/08870446.2022.2139830 36317294

[24] Palinkas LA, Horwitz SM, Green CA, Wisdom JP, Duan N, Hoagwood K. Purposeful Sampling for Qualitative Data Collection and Analysis in Mixed Method Implementation Research. Adm Policy Ment Health. 2015;42(5):533–44. 24193818 10.1007/s10488-013-0528-y PMC4012002

[25] Morgan DL, Krueger RA, King JA. The focus group guidebook. Thousand Oaks, CA, USA: Sage; 1998.

[26] Watkins DC. Rapid and Rigorous Qualitative Data Analysis: The “RADaR” Technique for Applied Research. Int J Qual Methods. 2017;16(1):1–9. 10.1177/1609406917712131

[27] Pinto RM, Park SE, Miles R, Ong PN. Community engagement in dissemination and implementation models: A narrative review. Implement Res Pract. 2021;2:2633489520985305. 37089998 10.1177/2633489520985305 PMC9978697

[28] Kegler MC, Halpin SN, Butterfoss FD, Wolfe SM. Evaluation methods commonly used to assess effectiveness of community coalitions in public health: Results from a scoping review. In: Price AW, Brown KK, editors. Evaluating Community Coalitions and Collaboratives New Directions for Evaluation 165. 2020. p. 139–57.

[29] Medicine NAo. Assessing Meaningful Community Engagement n.d. Available from: https://nam.edu/programs/value-science-driven-health-care/assessing-meaningful-community-engagement/.

[30] Aguilar-Gaxiola S, Ahmed SM, Anise A, Azzahir A, Baker KE, Cupito A, et al. Assessing Meaningful Community Engagement: A Conceptual Model to Advance Health Equity through Transformed Systems for Health: Organizing Committee for Assessing Meaningful Community Engagement in Health & Health Care Programs & Policies. NAM Perspect. 2022;2022. 10.31478/202202c. 10.31478/202202c PMC9303007 35891775

[31] PCORI. Advancing the Science of Engagement PCORI Funding Announcement -- Cycle 1 2024 2024 Available from: https://www.pcori.org/funding-opportunities/announcement/advancing-science-engagement-pcori-funding-announcement-cycle-1-2024.

[32] Ilias MR, Zhang X, Stinson N, Carrington K, Huff E, Freeman N, et al. Establishing a Community Engagement Consultative Resource: A CEAL Initiative. Am J Public Health. 2024;114(S1):S22–4. 37733995 10.2105/AJPH.2023.307385 PMC10785170

